# Prolonged Antibiotics Versus Prolonged Anticoagulation: A Case Report of Libman-Sacks Endocarditis

**DOI:** 10.7759/cureus.83203

**Published:** 2025-04-29

**Authors:** Muhammad A Zaman, Muhammad Ovais Sohail, Ibrahim Sbeitan, Salah M Aldergash

**Affiliations:** 1 Internal Medicine, Conemaugh Memorial Medical Center, Johnstown, USA; 2 Oncology, University of Pittsburgh Medical Center, Johnstown, USA

**Keywords:** advanced pancreatic cancer, libman-sacks endocarditis, marantic endocarditis, nonbacterial thrombotic endocarditis (ntbe), verrucous endocarditis

## Abstract

Marantic endocarditis, also known as nonbacterial thrombotic endocarditis (NBTE), Libman-Sacks endocarditis, or verrucous endocarditis, is a rare, non-infectious endocarditis (IE) that primarily aﬀects the aortic and mitral valves. It is often underreported due to its subtle nonspecific presentation and close echocardiographic resemblance to infective endocarditis (IE). Substantial NBTE diﬀerentials include cardiac tumors, IE, and prior residual lesions. Echocardiography, clinical evaluation, and other alternative imaging modalities, such as cardiac CT or PET/CT, are essential for comprehensive assessment. Treatment options primarily focus on managing the underlying condition and preventing thromboembolic events. As NBTE is characterized by sterile vegetations on cardiac valves and is not caused by an infectious agent, antibiotics have no role in treating NBTE. Anticoagulation is a critical component of treatment in patients with NBTE. However, the recommended duration of anticoagulation is not known and is a case-based decision. The American College of Chest Physicians guidelines suggest that patients with NBTE and systemic or pulmonary emboli should be treated with full-dose intravenous unfractionated heparin or subcutaneous low molecular weight heparin. It is suggested that anticoagulation should continue until the vegetation resolves (median of 11 months) or for at least one to two years to mitigate the systemic embolic risks.

## Introduction

Marantic endocarditis, also known as nonbacterial thrombotic endocarditis (NBTE), Libman-Sacks endocarditis, or verrucous endocarditis, is a non-infectious endocarditis (IE). The low incidence of NBTE is multifactorial, which could be explained by its close resemblance to IE. It is increasingly recognized due to better imaging modalities. In a contemporary 20-year cohort study at the Cleveland Clinic, NBTE was identified in 42 patients out of 600,577 transthoracic echocardiograms (TTEs) and 89,264 transesophageal echocardiograms (TEEs) [1]. Additionally, a necropsy series reported an incidence of 1.6% in the adult autopsy population [2]. NBTE closely mimics IE. Malignancies, especially mucin-producing adenocarcinomas, are significantly associated with NBTE. The pathogenesis is characterized by a multifactorial interplay of elements, including endothelial injury, hypercoagulability, and immune complex deposition, which are frequently observed in patients diagnosed with cancer. It is pertinent to mention that a hypercoagulable state predisposes thrombotic vegetations on the heart valves rather than actual IE. This diﬀerentiation is essential as treatment diﬀers significantly in both situations. Overall, the management of NBTE requires a multidisciplinary approach involving cardiologists for echocardiographic imaging assessment of NBTE, oncologists for cancer management plans, rheumatologists to rule out other autoimmune diseases contributing to NBTE, and sometimes cardiac surgeons for surgical resection of the vegetation. This collaborative strategy is essential to optimize patient outcomes and address the multifaceted nature of the condition. According to the American College of Chest Physicians, in patients with NBTE, it is suggested that patients use full-dose intravenous unfractionated heparin or subcutaneous low molecular weight heparin over no anticoagulation [3]. Although warfarin is not the primary recommended anticoagulant, it may be used due to its low cost and the patient’s preference for oral medication in selected cases. In this case report, we present a case of a 68-year-old female with recently diagnosed pancreatic adenocarcinoma leading to NBTE. 

## Case presentation

Presenting complaint

A 68-year-old woman presented to the emergency department (ED) with an acute onset of right-sided visual field loss and intermittent headaches for one day. Over the past few weeks, she had experienced an unintentional weight loss of 12 pounds, worsening fatigue, generalized abdominal pain, and a decreased appetite. The past medical history is significant for deep vein thrombosis, gastroesophageal reflux disease, hyperlipidemia, and a recently diagnosed pancreatic mass. On arrival, the patient’s vital signs were as follows: blood pressure of 113/67 mmHg, heart rate of 114 beats per minute, temperature of 96.8 °F, respiratory rate of 18 breaths per minute, and oxygen saturation within normal limits on room air. She displayed no acute distress and denied any fever, chills, chest pain, or dyspnea. The physical examination was unremarkable, except for the neurological examination, which revealed a right homonymous hemianopia. The National Institutes of Health Stroke Scale score was 2. There were no murmurs on chest auscultation.

Initial workup

**Figure 1:**
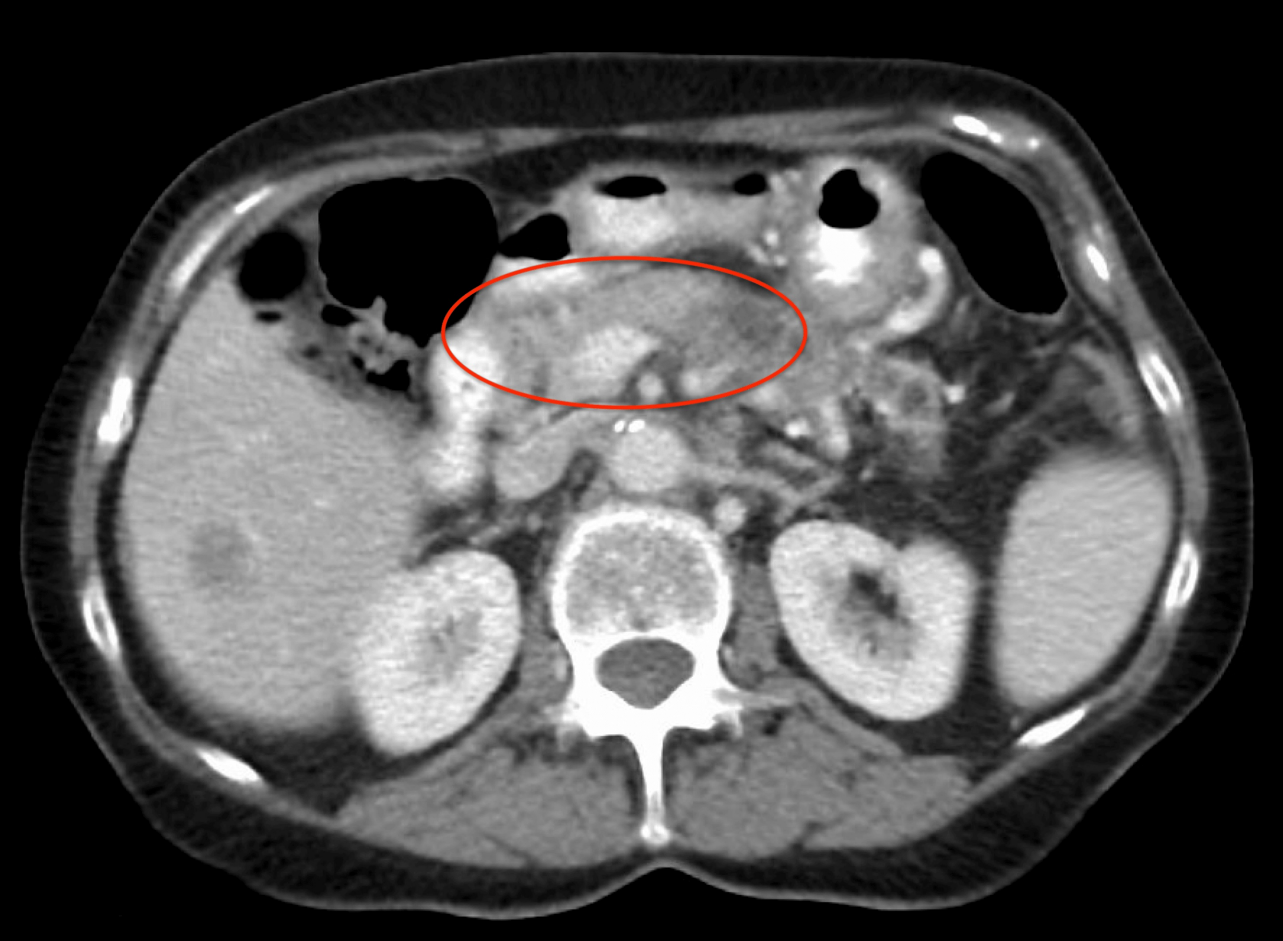
CT of abdominal and pelvis, revealing pancreatic mass

Table 1 presents the initial laboratory workup at the time of presentation. A CT scan of the head showed a subacute infarction in the left occipital lobe and a right frontal subcortical hypodensity, consistent with an ischemic stroke. The patient was outside the therapeutic window for thrombolysis, and no large vessel occlusion was found to warrant thrombectomy. A CT scan of the abdomen, conducted two weeks earlier, had shown findings consistent with pancreatic cancer, including a pancreatic mass suspicious for malignancy, metastatic liver lesions, omental caking, and mild ascites. Figure 1 depicts the newly diagnosed pancreatic mass. The patient also exhibited elevated levels of carcinoembryonic antigen (CEA), carbohydrate antigen (CA) 19-9, and CA 125, which were measured as part of the initial cancer workup to aid in monitoring disease progression and treatment response.

**Table 1:** Initial laboratory results at presentation

Laboratory Test	Result	Reference Range
White Blood Cell Count	7.6x10⁹/L	4.0-11.0x10⁹/L
Red Blood Cell Count	4.06x10¹²/L	4.2-5.9x10¹²/L
Hemoglobin	12.8 g/dL	12.0-16.0 g/dL
Hematocrit	38%	36-46%
Platelet Count	199x10⁹/L	150-450x10⁹/L
Prothrombin Time	12.5 sec	11-14 sec
International Normalized Ratio	1.0	0.8-1.2
Partial Thromboplastin Time	30 sec	25-35 sec
Carcinoembryonic Antigen	27.29	<5.0 ng/mL
Cancer Antigen 19-9	Elevated	<37 U/mL
Cancer Antigen 125	302	<35 U/mL
Sodium	132 mmol/L	135-145 mmol/L
Potassium	3.9 mmol/L	3.5-5.1 mmol/L
Chloride	101 mmol/L	96-106 mmol/L
Bicarbonate	23 mmol/L	22-28 mmol/L
Blood Urea Nitrogen	11 mg/dL	7-20 mg/dL
Creatinine	0.7 mg/dL	0.6-1.3 mg/dL
Glucose	87 mg/dL	70-100 mg/dL
Total Bilirubin	0.8 mg/dL	0.1-1.2 mg/dL
Direct Bilirubin	0.2 mg/dL	0.0-0.3 mg/dL
Aspartate Aminotransferase	22 U/L	10-40 U/L
Alanine Aminotransferase	18 U/L	7-56 U/L
Alkaline Phosphatase	75 U/L	40-130 U/L
D-dimer	0.9 μg/mL	<0.5 μg/mL
Fibrinogen	420 mg/dL	200-400 mg/dL
Lactate Dehydrogenase	310 U/L	140-280 U/L
C-reactive Protein	5.5 mg/dL	0.0-0.8 mg/dL
Erythrocyte Sedimentation Rate	14 mm/hr	0.0-30 mm/hr

Initial ED management

Day One of Admission

Initial management included aspirin 81 mg daily and atorvastatin 40 mg, telemetry monitoring, and plans for further evaluation, including MRI of the brain, lower extremity doppler ultrasound, CT angiography of the neck, and an echocardiogram with a bubble study to investigate a possible cardioembolic source.

Days Two to Five of Admission

**Figure 2:**
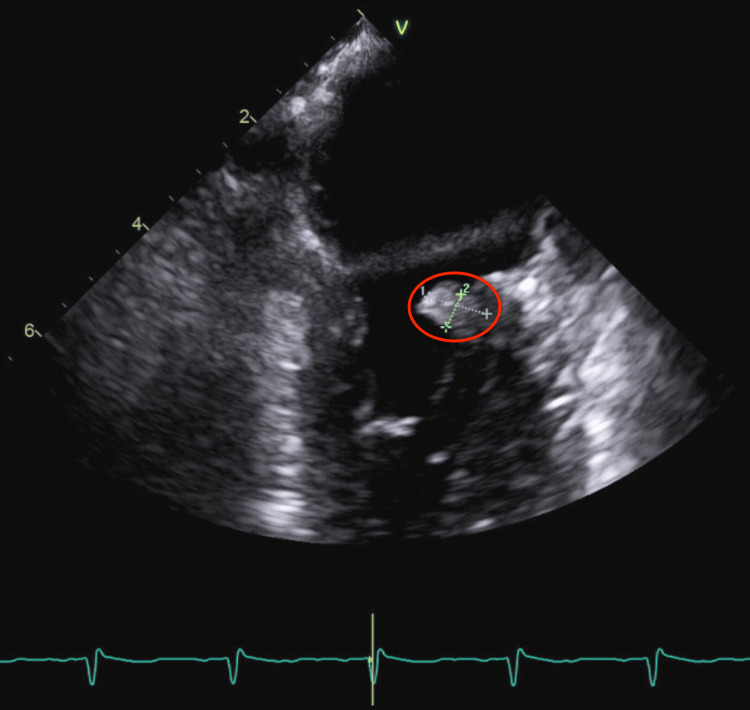
TEE, revealing a mitral valve vegetation TEE, transesophageal echocardiography

**Figure 3:**
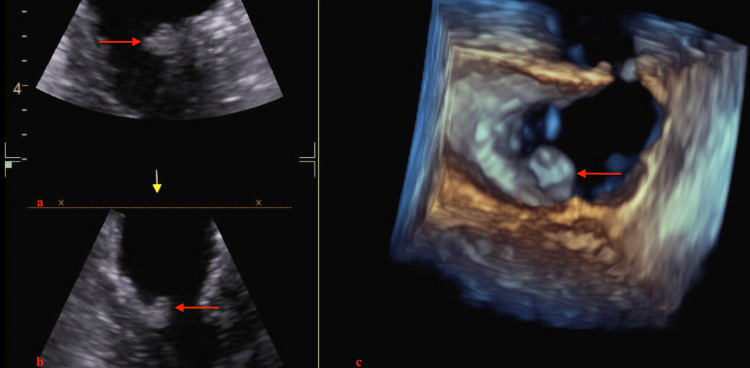
TEE (a, b) Mid-esophageal two-chamber views revealing mitral valve vegetation (indicated by red arrows). (c) Reconstructed 3D view showing mitral valve vegetation (red arrow). TEE, transesophageal echocardiography

The brain MRI revealed a subacute infarction in the left occipital lobe and multiple subcentimeter infarctions in both cerebral hemispheres, the cerebellum, and subcortical regions, indicating a diffuse embolic process. The endoscopic ultrasound (EUS) confirmed a pancreatic body mass measuring 20x18 mm, along with multiple liver metastases and malignant ascites. Fine needle aspiration of a hepatic lesion provided preliminary results that raised suspicion of adenocarcinoma. The cancer was staged as T2 N1 M1, consistent with stage IV disease. Histopathology indicated tumor cells expressing mucicarmine, CK7, CEA, CA 19-9, and p53. TEE revealed a mobile, irregular mass measuring 8 5 mm on the atrial side of the posterior mitral valve leaflet, with no significant mitral regurgitation, suggestive of infective endocarditis (IE) (TTE did not reveal vegetation). Figure 2 and Figure 3 display the TEE findings. 

Serial blood cultures were negative, and no fever or systemic infections were present. The findings from the TEE, combined with the patient’s pancreatic cancer, which made her more prone to a hypercoagulable state, led to a diagnosis of NBTE. The patient was initially started on a heparin infusion on day three and later transitioned to enoxaparin 60 mg twice daily for discharge at home on day seven of admission.

Follow-up

She started chemotherapy within one week of being discharged from the hospital. She could not tolerate the chemotherapy and opted for hospice care. The patient passed away after three weeks in hospice care.

## Discussion

NBTE, marantic endocarditis, or Libman-Sacks endocarditis is a rare condition, with prevalence estimates ranging from 0.3% to 9% in the autopsy reports of the adult population, often linked with malignancy or systemic inflammatory states [4,5]. It involves sterile fibrin and platelet thrombi that form on cardiac valves without associated infection [6]. It is often diagnosed in advanced cancer patients driven by release due to hypercoagulable states triggered by tumor-secreted cytokines such as tissue factor [7]. These procoagulant factors promote thrombin and fibrin formation. The immune complex depositions in autoimmune conditions can also contribute to endothelial injury.

These thrombi pose a significant risk for systemic embolization, leading to ischemic strokes and other complications. The in-hospital mortality rate for NBTE is notably high, reported at 30% in some studies [8]. Among patients with malignancy-associated NBTE, the prognosis is abysmal, with a mortality rate of 87% and an average time from diagnosis to death of 34 days [9]. The clinical implications of NBTE are profound, as it can lead to recurrent embolic phenomena affecting multiple organ systems, including the brain, kidneys, and extremities. Cerebrovascular accidents are a common manifestation, and patients often present with sudden neurological deficits. Diagnosis involves identifying valve masses on echocardiograms without positive blood cultures or any other signs of infections. TEE remains the gold standard for identifying valve lesions, providing superior sensitivity to transthoracic imaging [10]. Although TEE is not an absolute requirement, in cases when clear vegetations are seen on TTE, it is preferred due to its superior sensitivity in detecting small, mobile, and posteriorly located vegetations compared to TTE. No biopsy was done of the vegetation in our case. Biopsy of vegetation in NBTE is generally not recommended due to procedural risks of potential embolization and valve damage. The different societal guidelines recommend using less invasive diagnostic modalities including TEE, cardiac MRI, and PET/CT scans. The biopsy is only considered when imaging and clinical findings are inconclusive, and the risk-benefit ratio is favorable.

Non-bacterial vegetations on cardiac valves in NBTE can closely resemble those found in IE on imaging and clinical presentation. Both conditions may demonstrate valvular masses, systemic embolic phenomena, and new murmurs or heart failure features. Using the 2023 Duke-International Society for Cardiovascular Infective Diseases Criteria for Infective Endocarditis, we formulated Table 2, which suggested possible IE [11]. However, the distinguishing factor lies in the sterility of NBTE vegetations, as evidenced by consistently negative blood cultures and the absence of clinical signs of infection. This overlap in presentation can lead to diagnostic uncertainty, especially when patients with NBTE present with fever or elevated inflammatory markers, which are often mistakenly attributed to an underlying infection. This mimicry between NBTE and IE underscores the importance of a meticulous diagnostic approach. Overreliance on findings without taking into context the complete clinical assessment may result in misdiagnosis. 

**Table 2:** Duke's Criteria for IE Source/adapted from Fowler VG et al. DOI: 10.1093/cid/ciad271 [11]. Data subsetted to include only relevant information. IE, infective endocarditis

Criteria	Findings	Present in Patient
Major Criteria	Positive blood cultures for typical organisms	No
	Evidence of endocardial involvement on echocardiogram	Yes (mitral valve vegetation)
Minor Criteria	Predisposing heart condition or intravenous drug use	No
	Fever ≥38°C	No
	Vascular phenomena (e.g., emboli, Janeway lesions)	Yes (embolic stroke)
	Immunologic phenomena (e.g., glomerulonephritis)	No
	Microbiological evidence not meeting major criterion	No

The treatment of both conditions is significantly different, and physicians cannot afford to misinterpret NBTE with IE and vice versa. Extreme caution is advised before making treatment decisions for NBTE. While IE requires prompt initiation of targeted antibiotic therapy, NBTE requires prolonged anticoagulation [12]. Additionally, misdiagnosing NBTE as IE and prematurely starting antibiotics can lead to unnecessary antibiotic overuse, resistance, adverse effects, and increased healthcare costs. More critically, this delay in recognizing NBTE postpones anticoagulation, the cornerstone of its management, increasing the risk of recurrent embolic events.

The assessment of bleeding risk and the evaluation of the risk-benefit balance of anticoagulation therapy are critical. The American College of Chest Physicians guidelines highlight that advanced age, previous bleeding, renal failure, liver failure, thrombocytopenia, and metastatic cancer significantly increase bleeding risk [13]. The American Society of Clinical Oncology recommends caution with direct factor Xa inhibitors in patients with gastrointestinal and genitourinary malignancies due to a higher risk of mucosal bleeding [14]. The decision to continue anticoagulation in NBTE should weigh the high risk of thromboembolic events against the potential for bleeding. Table 3 represents key highlights of clinical or imaging distinctions that aid in the differentiation of IE versus NBTE. 

**Table 3:** The key clinical or imaging distinctions in the differentiation of IE and NBTE NBTE, nonbacterial thrombotic endocarditis; IE, infective endocarditis

	IE	NBTE
Clinical Distinctions	Presents with fever, positive blood cultures, and signs of systemic infection	Presents with embolic events, particularly cerebrovascular accidents
Modified Duke Criteria	+ve	-ve
Associations	Congenital heart disease, prosthetic valves, or intravenous drug use	Hypercoagulable states, malignancies, and autoimmune disorders such as systemic lupus erythematosus and antiphospholipid syndrome
Echocardiography	Often larger, more irregular vegetations, and may be associated with abscesses, pseudoaneurysms, or leaflet perforations	Typically smaller, more homogeneous, and less likely to cause significant valvular dysfunction

As the management of NBTE focuses on prolonged anticoagulation to prevent further embolic events and address the underlying condition, often a malignancy, in our case, the patient was started on enoxaparin, a low molecular weight heparin. While anticoagulation can reduce the risk of further thromboembolic events, it does not resolve existing valvular lesions. Anticoagulation therapy can lead to improved survival or stabilization in patients with cancer-associated NBTE by reducing the risk of thromboembolic events, as supported by current guidelines and clinical studies [15]. In patients with NBTE, advanced imaging modalities and multidisciplinary discussions are often necessary to avoid misdiagnosis and ensure that management strategies are appropriately tailored to the underlying etiology. There are no official guidelines for monitoring, and this is usually done through an individualized approach. Indefinite anticoagulation if the cancer is not controlled, and even if successfully controlled, re-evaluate the need for anticoagulation. 

## Conclusions

This case highlights the importance of maintaining a high index of suspicion for NBTE in patients with certain risk factors. One of the crucial differentials is IE; clinicians must be able to differentiate both while considering the clinical context. Specifically, misdiagnosing NBTE as IE can lead to unnecessary antibiotic use while misdiagnosing IE as NBTE can delay life-saving antimicrobial therapy.

Early recognition and appropriate management, including echocardiographic evaluation and anticoagulation, are crucial for preventing complications such as valvular dysfunction, heart failure, and systemic embolization. It is pertinent to keep in mind that the high risk of recurrent embolic events (10-15%) remains high despite being on anticoagulation. The main gaps in understanding NBTE in cancer patients are related to its pathogenesis, risk stratification, and optimal anticoagulation strategies. Addressing these gaps through targeted research could significantly improve patient outcomes.
